# Molecular neuroanatomy of anorexia nervosa

**DOI:** 10.1038/s41598-020-67692-1

**Published:** 2020-07-10

**Authors:** Derek Howard, Priscilla Negraes, Aristotle N. Voineskos, Allan S. Kaplan, Alysson R. Muotri, Vikas Duvvuri, Leon French

**Affiliations:** 1 Campbell Family Mental Health Research Institute, Centre for Addiction and Mental Health, Toronto, Canada; 2 Krembil Centre for Neuroinformatics, Centre for Addiction and Mental Health, Toronto, ON Canada; 3 Department of Pediatrics, School of Medicine, University of California San Diego, La Jolla, CA USA; 4 Institute for Medical Science, University of Toronto, Toronto, Canada; 5 Slaight Family Centre for Youth in Transition, Centre for Addiction and Mental Health, Toronto, ON Canada; 6 Department of Psychiatry, University of Toronto, Toronto, Canada; 7 Department of Pediatrics/Cellular and Molecular Medicine, University of California San Diego, La Jolla, CA USA; 8 Kavli Institute for Brain and Mind, University of California San Diego, La Jolla, CA USA; 9 Rady Children’s Hospital, San Diego, CA USA; 10 Department of Pediatrics and Psychiatry, School of Medicine, University of California San Diego, La Jolla, CA USA

**Keywords:** Data integration, Transcriptomics, Neuroscience, Brain

## Abstract

Anorexia nervosa is a complex eating disorder with genetic, metabolic, and psychosocial underpinnings. Using genome-wide methods, recent studies have associated many genes with the disorder. We characterized these genes by projecting them into reference transcriptomic atlases of the prenatal and adult human brain to determine where these genes are expressed in fine detail. We found that genes from an induced stem cell study of anorexia nervosa cases are expressed at higher levels in the lateral parabrachial nucleus. Although weaker, expression enrichment of the adult lateral parabrachial is also found with genes from independent genetic studies. Candidate causal genes from the largest genetic study of anorexia nervosa to date were enriched for expression in the arcuate nucleus of the hypothalamus. We also found an enrichment of anorexia nervosa associated genes in the adult and fetal raphe and ventral tegmental areas. Motivated by enrichment of these feeding circuits, we tested if these genes respond to fasting in mice hypothalami, which highlighted the differential expression of *Rps26* and *Dalrd3*. This work improves our understanding of the neurobiology of anorexia nervosa by suggesting disturbances in subcortical appetitive circuits.

## Introduction

Anorexia nervosa is a complex eating disorder that primarily occurs in women with onset often occurring during adolescence. While a portion of anorexia nervosa patients sustain a full recovery, a substantial fraction suffer from protracted partial recovery or are afflicted with a chronic disease course^1^. Currently, there are few effective treatments, and the standard-of-care is broadly aimed at psychological and nutritional recovery^2,3^. Specifically, for youth, family-based treatment has been shown to be beneficial^4^. Due to self-starvation and suicide, anorexia nervosa is consistently reported to be among psychiatric disorders with the highest all-cause mortality ratios^5,6^.

Our understanding of the neuroanatomical circuits involved in anorexia nervosa is limited. MRI studies of people affected with the illness are only able to examine large structures, and such studies find broad volume reductions and white matter alterations^7–9^. Diffusion tensor imaging studies have found changes in several white matter tracts^10^. Due to the coarse resolution of MR images, these studies cannot detect structural or functional changes at the microcircuit level. In addition, it is difficult to identify structural alterations that may underlie the disorder due to a lack of studies initiated prior to the onset of symptoms. While subjects with anorexia nervosa are noted to have widespread cortical thinning and decreased volume, recent studies report that measured alterations in brain structure reflect changes in nutritional status and that weight restoration can rapidly reverse these changes in younger patients^11–13^. Regional analyses have found stronger differences in reward and somatosensory regions. The identification of the reward areas was hypothesized to mark aberrant reward responses to food and disrupted body perception^14^. In contrast, animal experiments have deciphered subcortical circuits that control appetite and feeding behaviours^15–19^. We note that these animal experiments are focused on true anorexia (loss of appetite) and do not mimic the complex symptoms of anorexia nervosa. Nonetheless, the neural circuits found in mice may inform studies of patients with the disorder.

Importantly, anorexia nervosa has a strong genetic basis. Twin-based heritability is estimated to range from 48 to 74%^20^ and genetic studies are starting to identify candidate genes. Specifically, four past studies have used genome-wide scans to associate genes and genetic variants with the disorder. First, a transcriptomic study that compared induced neural stem cells of anorexia nervosa patients and controls identified hundreds of differentially expressed genes^21^. This study highlighted differential expression of the tachykinin 1 receptor and suggested that an abnormal tachykinin neuropeptide signalling pathway might underlie the disorder. At the genetic level, whole-exome sequencing identified damaging rare variants that are associated with disordered eating^22^. These variants were enriched for neuropeptide signalling genes. The first meta-analysis of common genetic variants identified the first genome-wide significant locus for anorexia nervosa (rs4622308) that overlaps with six genes^23^. Using a larger combined cohort, the most recent genome-wide association study (GWAS) meta-analysis identified eight significant risk loci^24^. These loci implicate the nearby genes through genomic proximity, but more evidence is needed to determine if they are causal. Through integration with other GWAS results, the common variant studies revealed genetic correlations with metabolic traits. While all of these studies use genome-wide assays, the approaches capture different aspects. The exome study sought to find rare genetic variants with high penetrance, while genome-wide association studies identify common variants with small effects. In contrast, differential expression of genes in induced neural stem cells would represent the downstream effects of genetic risk^25,26^. While these genomic results are limited in sample size and validations, they have provided the first sets of candidate causal genes for anorexia nervosa to date.

Previous studies have attempted to determine which brain regions and cell types most express genes associated with the disorder. Negraes and colleagues used the BrainSpan Atlas of the developing human brain to characterize the expression of their top gene, *TACR1*. They found it was expressed highly in the striatum during adolescence^21^. To narrow the genes linked to the first genome-wide significant locus, Duncan et al. tested for expression quantitative trait loci in human tissues and differential expression in fasted male mice, but no statistically significant relationships were found^23^. To broadly characterize the common variant associations, Watson et al. used partitioned heritability analysis to determine enrichment for genes highly expressed in a broad range of mouse cell-types and human tissues. This specificity analysis identified brain tissues and neurons in the striatum and hippocampus^24^. These first analyses suggest that more in-depth characterization of these genes may more precisely indicate cell types and neural circuits that may be particularly relevant to the disorder.

**Figure 1:**
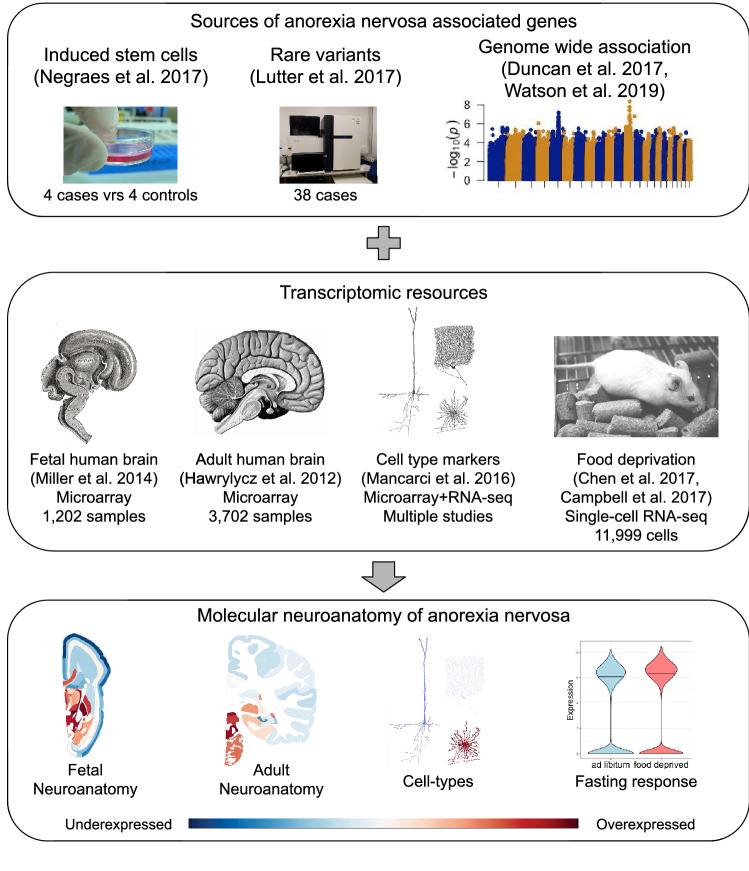
Overview of the study. Genes associated with anorexia nervosa (top) are from several sources. For these studies, varying amounts of genes were associated: Negraes et al. (361 genes), Lutter et al. (186 genes), Duncan et al. (6 genes), and Watson et al. (107 genes). These studies highlighted neuropeptide signalling genes and genetic correlations with metabolic traits. After filtering, these genes are characterized in several genome-wide expression datasets (middle) to provide locations, cell types, and fasting expression differences (bottom). These resources are obtained from microarray and RNA sequencing assays of gene expression profiles obtained from the human and mouse brain. Images are from the cited publications, and Wikimedia Commons (Gray's Anatomy by Henry Vandyke Carter [public domain]; Sobotta's Human Anatomy 1908 [public domain]; users kaibara87 [changes: converted to grayscale, Creative Commons Attribution 2.0 Generic license, https://creativecommons.org/licenses/by/2.0/deed.en], Rama [ Creative Commons Attribution-Share Alike 2.0 France license, https://creativecommons.org/licenses/by-sa/2.0/fr/deed.en], and Konrad Förstner [ Creative Commons CC0 1.0 Universal Public Domain Dedication, https://creativecommons.org/publicdomain/zero/1.0/deed.en]).

These anorexia nervosa associated genes do not provide a direct link to a specific brain region or cell type because they were derived from fibroblasts or DNA. To better characterize the molecular neuroanatomy of anorexia nervosa, we first examined these genes in transcriptomic atlases of the human brain (Fig. 1). We hypothesized regions in subcortical appetitive circuits and existing targets for brain stimulation would be enriched for higher expression of the associated genes. We chose the Allen Brain Atlases for this analysis due to their comprehensive coverage of fine subcortical nuclei. These atlases are of normal brains and can provide spatial insight into molecular events that underlie the disorder. While the examination of postmortem brains of cases is possible, it is difficult to determine which genes are perturbed by malnutrition or are causal. To gain a cell-type perspective that is not provided in the transcriptomic atlases of bulk brain tissue, we tested for enrichment of cell-type markers in the anorexia nervosa associated gene lists. Mirroring our focus on subcortical feeding circuits, we tested for expression changes in response to fasting in mice hypothalami. Broadly, we sought to leverage existing transcriptomic resources to better understand genes recently linked to anorexia nervosa.

## Methods

### Adult human brain gene expression data

The Allen Human Brain Atlas provides a comprehensive transcriptional landscape of six normal human brains^27^. Complete microarray gene expression datasets were downloaded from the Allen Human Brain Atlas data portal (https://human.brain-map.org/static/download/). These datasets were obtained from six individuals (five males, one female), with age ranging from 24 to 57 years. Custom 64 K Agilent microarrays were used to assay genome-wide expression in 3,702 spatially-resolved samples (232 named brain regions). The Allen Institute normalized the data with a multistep process that adjusted for array-specific biases, batch, and dissection method. Full details of the procedures used by the Allen Institute researchers are available in the Allen Human Brain Atlas technical white paper (https://help.brain-map.org/display/humanbrain/Documentation). Anatomical images used in the figures were obtained from the Allen Human Brain Reference Atlas^28^.

### Prenatal human gene expression data

Similar to the Allen Human Atlas, this transcriptomic atlas of the normal mid-gestational human brain provides a brain- and genome-wide reference^29^. Complete microarray gene expression datasets were downloaded from the Brainspan website (https://www.brainspan.org/static/download.html). Datasets were obtained from 4 intact mid-gestational human brains that passed several exclusion criteria (15–21 post-conception weeks, 3 females). The same custom 64 K Agilent microarrays that were used for the adult atlas were used to assay expression in the 1,203 spatially-resolved samples (516 named brain regions). Details of the procedures used by the Allen Institute researchers are available in the Brainspan Atlas of the Developing Human Brain technical white paper (https://help.brain-map.org/display/devhumanbrain/Documentation). Anatomical images used in the figures were obtained from the Allen Prenatal Human Brain Reference Atlas^29^.

### Microarray gene expression data processing

Microarray probes were re-annotated using the Re-Annotator mRNA reference annotations to increase the total number of annotated probes. Re-Annotator uses a customized reference sequence database to identify the positions of gene expression array probe sequences^30^. This tool removes uninformative probes, updates probe annotations and improved the total of probes used for our downstream analysis from 48,170 probes used for 20,737 genes using the default probes annotations to 54,966 probes used for 20,884 gene expression measurements. For each donor, samples mapping to the same-named brain region were mean averaged to create a single expression profile for each region. Analogous named brain regions of both hemispheres were not distinguished because no significant differences in molecular architecture have been detected between the left and right hemispheres^29^. Expression levels of the 58,692 probes were then summarized by mean averaging for each of 20,869 gene transcripts. Gene expression values were next converted to ranks within a named brain region and z-score normalized across brain regions. Z-scores for each donor were then averaged across donors to obtain a single gene by region reference matrix of expression values.

### Regions of interest

In addition to brain-wide analyses that consider all regions, we focused on brain regions and circuits previously associated with anorexia nervosa and feeding behaviour. We examined expression profiles of sites from deep brain stimulation studies: the nucleus accumbens and the subgenual region of the anterior cingulate cortex (Brodmann area 25)^31–34^. Specifically, the related studies and regions are detailed in Table 1 of Sobstyl et al.^33^. The hypothalamus and ventral tegmental area provide two additional regions that have been named as potential sites^34^. Guided by experimental mouse studies of feeding, we focused on several additional regions: arcuate nucleus of the hypothalamus, raphe magnus, raphe obscurus, dorsal raphe, central nucleus of the amygdala, nucleus of the solitary tract and the parabrachial nucleus^35^. Additional studies linking these regions are provided in Supplement Table 1. To align the Allen fetal atlas with the regions of interest we merged six regions to form an expression profile for the subgenual cingulate cortex and two regions for the central amygdala (Supplement Table 2). For the remaining regions, the names given match the labels used in the Allen Atlases.

### Brain region enrichment analysis

To calculate enrichment for the gene sets of interest within a brain region, z-scores in the processed expression matrices are ranked within a region, with high ranks marking specific expression and low for depleted expression. Our genes of interest were then projected into these ranked lists for each region. The area under the receiver operating curve (AUROC) statistic was used to quantify if the anorexia nervosa associated genes are more specifically expressed (ranked higher) in this sorted list of genes for a specific region or are randomly distributed within the sorted list. This threshold-free non-parametric measure is commonly used for gene set enrichment testing^36^. For a given region, the AUROC for an anorexia nervosa associated gene set is the probability that a gene from that set will be found first in the genome-wide ranking compared to the rest of the genome. In this context, AUROC > 0.5 means the anorexia nervosa associated genes have specific expression in a region. For AUROC < 0.5, the reverse is true, with a bias toward lower relative expression in a specific region. The Mann–Whitney U test was used to test the statistical significance of an AUROC statistic. Benjamini–Hochberg false discovery rate procedure was used to correct for testing of multiple brain region tests within a dataset.

### Anorexia Nervosa associated gene lists

#### iPSC gene list

The initial discovery gene set contained 361 differentially expressed genes obtained from iPSC-derived neurons from anorexia nervosa patients compared to controls (Negraes et al. 2016). Specifically, we used the gene symbols in Supplement Table 5. To improve integration with the Allen data, we mapped antisense and intronic transcripts to the symbol of the protein-coding gene by removing -AS and -IT suffixes. After these edits, 289 of 361 genes matched to the gene symbols of processed adult and fetal human brain gene expression datasets.

#### Watson et al. gene list

The GWAS meta-analysis of anorexia nervosa (16,992 cases and 55,525 controls) identified 8 genome-wide significant loci^24^. Supplemental Table 7 provided a list of 107 protein-coding genes annotated as proximal to the genome-wide significant loci. The majority of the annotated protein-coding genes (96 of 107) are in proximity to the first identified locus (rs9821797). We also examined the 76 genes prioritized via MAGMA gene-wise analysis, of which 60 genes overlap with the list of 107 protein-coding genes.

#### Whole exome sequencing lists

Genes containing rare variants associated with disordered eating, as determined by exome sequencing were obtained from Lutter et al.^22^. Specifically, Table S3 provided a list of 186 genes that harboured damaging variants in individuals with the restricting type of anorexia nervosa and Table S4 provided a list of 245 damaging variants in individuals with binge-eating episodes which was used as a comparison gene set to test specificity. For both of these gene lists, we applied a conservative significance threshold by calculating the Bonferroni corrected p-values with an estimate of 20,000 tested genes. After applying this filter, 51 genes associated with restricted eating and 80 genes associated with binge-purge eating disorders remained.

### Mouse cell type-specific marker genes

Marker genes for major cell types were obtained from the NeuroExpresso database that performed a cross-laboratory analysis of several mouse gene expression studies^37^. The marker sets were obtained from https://github.com/oganm/neuroExpressoAnalysis. In addition to the combined set, marker genes from cortical, brainstem, and amygdala analyses were used (based on the regions of interest).

### Gene expression responses to food deprivation in mice

Two mouse datasets were used to determine which of the anorexia nervosa associated genes are differentially expressed in response to food deprivation^38,39^. Both performed single-cell RNA sequencing to characterize transcriptional changes after food deprivation in the mouse hypothalamus.

The Chen et al. dataset assayed the transcriptome with single-cell RNA sequencing in over 14,000 cells from the mouse hypothalamus (adult female B6D2F1 mice)^38^. The “Cells.Expresssion.Matrix.log_tpm + 1” file from GSE87544 was used. This file contains expression values for 23,284 genes and 14,437 cells. We filtered for genes with human homologs in the homologene database, resulting in 16,155 genes ^40^. Genes with no expression in all cells were removed, leaving 15,217. Only cells from batch 1 and 2 were used because they contained data from both food-deprived and control animals, leaving 10,983 cells. The food-deprived animals were given only water for a 24 h period.

The Campbell et al. dataset contains gene expression profiles of over 20,000 cells from the arcuate hypothalamus and median eminence (transgenic with C57BL6/J background)^39^. Summarized gene expression profiles were obtained from the file named “GSE93374_Merged_all_020816_BatchCorrected_LNtransformed_doubletsremoved_Data.txt.gz”. This file contains expression values for 19,743 genes and 20,922 cells. To be consistent with the Chen dataset, we filtered these cells for those from female mice and only used batch 6, which also compared fasted (24 h) to normal mice (ad libitum chow-fed). After filtering out genes without human homologs and those without any variance in expression, 14,507 remained for 1,016 cells.

The Mann–Whitney U test was used to test for differential expression between the food-deprived or hunger conditions. This method has been shown to be adequate for testing differential expression in single-cell studies with simple designs^41^. For each gene, the three dataset/batches were tested separately (13,208 mouse genes in both datasets). Fisher’s method was used to combine these three p-values into single meta p-values for up- and down-regulation of expression after 24 h of food deprivation.

### Availability

Scripts, supplementary tables, and data files for reproducing the analyses are available online at https://github.com/derekhoward/molecular_AN and 10.6084/m9.figshare.7115693.

## Results

Our study begins by first comparing the anorexia nervosa associated gene lists to describe agreement across the four sources. To characterize the molecular neuroanatomy of anorexia nervosa, we next detail regional expression enrichment of the anorexia nervosa associated genes in large expression atlases. We perform these analyses using atlases of both the prenatal and adult brain to provide insight into patterns in early development and adulthood. We examine four sources of anorexia associated genes and provide a regional summary. To gain a cellular perspective, we next tested for enrichment of cell-type markers in the lists. Finally, we present our results of testing if the anorexia nervosa associated genes are enriched for differential expression in fasted mice as an exploratory analysis.

### Overlaps between anorexia nervosa associated gene lists

Across the four anorexia nervosa associated gene sets, four genes overlap between the Lutter damaging variants and Negraes iPSC derived gene set (*TNFRSF10A*, *SCGB1A1*, *IFIT3*, and *GIPC3*; hypergeometric test, *p* < 0.02). In contrast, only one gene overlaps with the binge eating associated genes identified by Lutter et al. (*BDNF*), and one appears in the protein-coding genes identified in the Watson et al. GWAS (*AMT*). The six genes overlapping with the rs4622308 locus did not appear in the Lutter, Watson, or Negraes sets. To characterize brain-wide neuroanatomical expression patterns, we used the Negraes list because it is the largest, providing the most statistical power to detect regions with specific enrichment. We then tested for particular regions of interest with the smaller sets obtained from the genetic studies.

### Negraes iPSC gene list: regional enrichment tests

**Figure 2:**
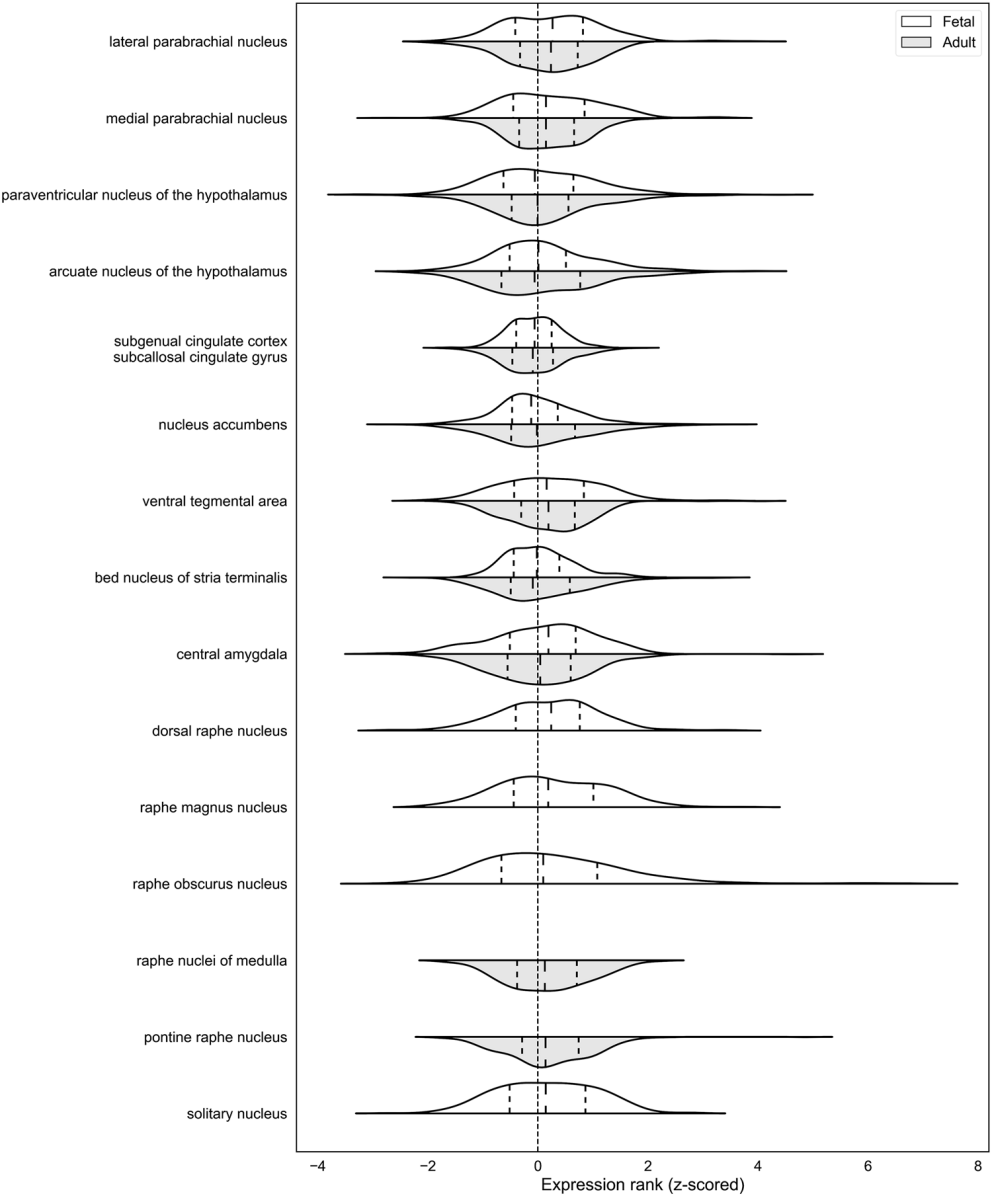
Violin plots showing the distributions of gene expression ranks of the set of AN associated genes derived from Negraes et al. Each violin is split to show the fetal (white) and adult (light grey) brain data. Dashed vertical lines within the violins mark expression quartile borders.

**Figure 3:**
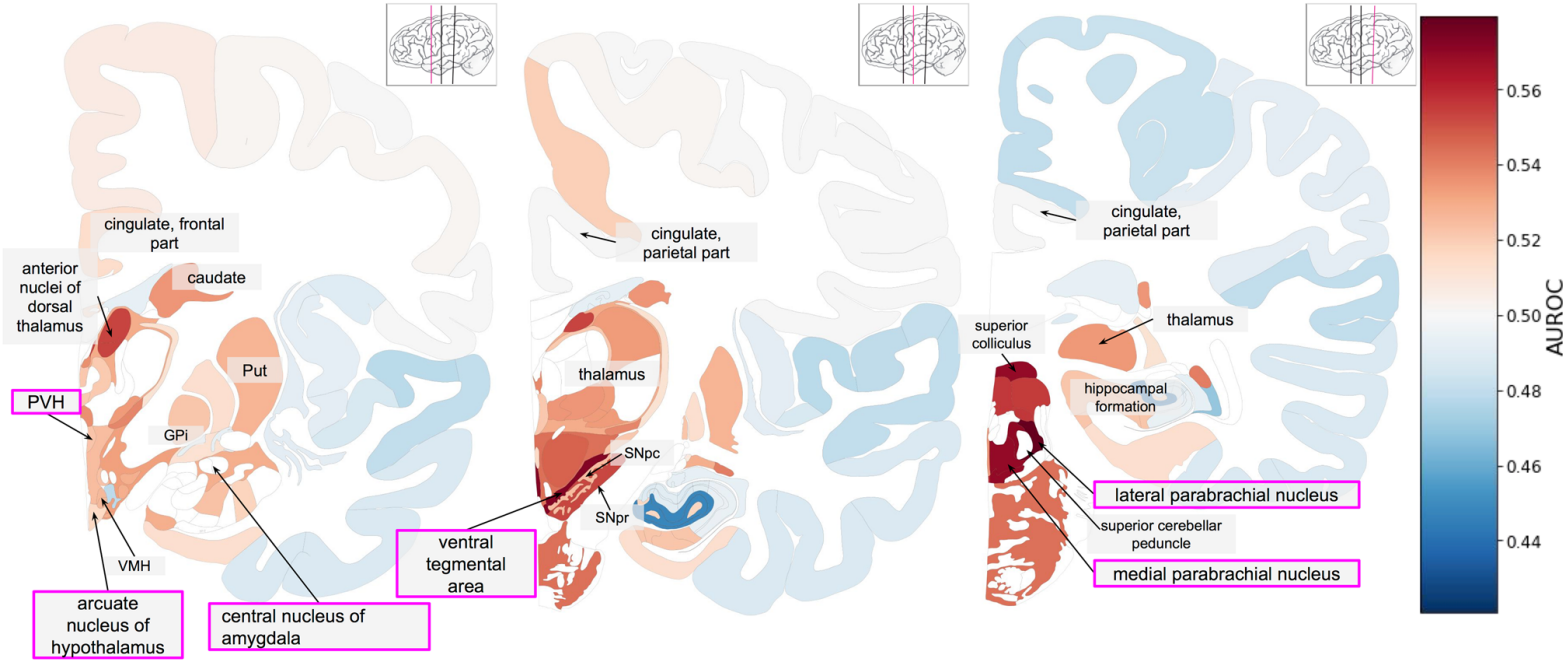
Anatomical maps showing aggregate gene expression patterns of the anorexia nervosa associated genes from Negraes et al. in the adult brain. Regions of interest are highlighted with purple boxes. The inset thumbnail marks the current slice in pink. AUROC values range from depleted expression in dark blue to enriched in dark red with missing values in white. (PVH: paraventricular nucleus of hypothalamus; GPi: globus pallidus, internal segment; Put: putamen; VMH: ventromedial hypothalamic nucleus; SNpr: substantia nigra pars reticulata; SNpc: substantia nigra pars compacta).

**Figure 4:**
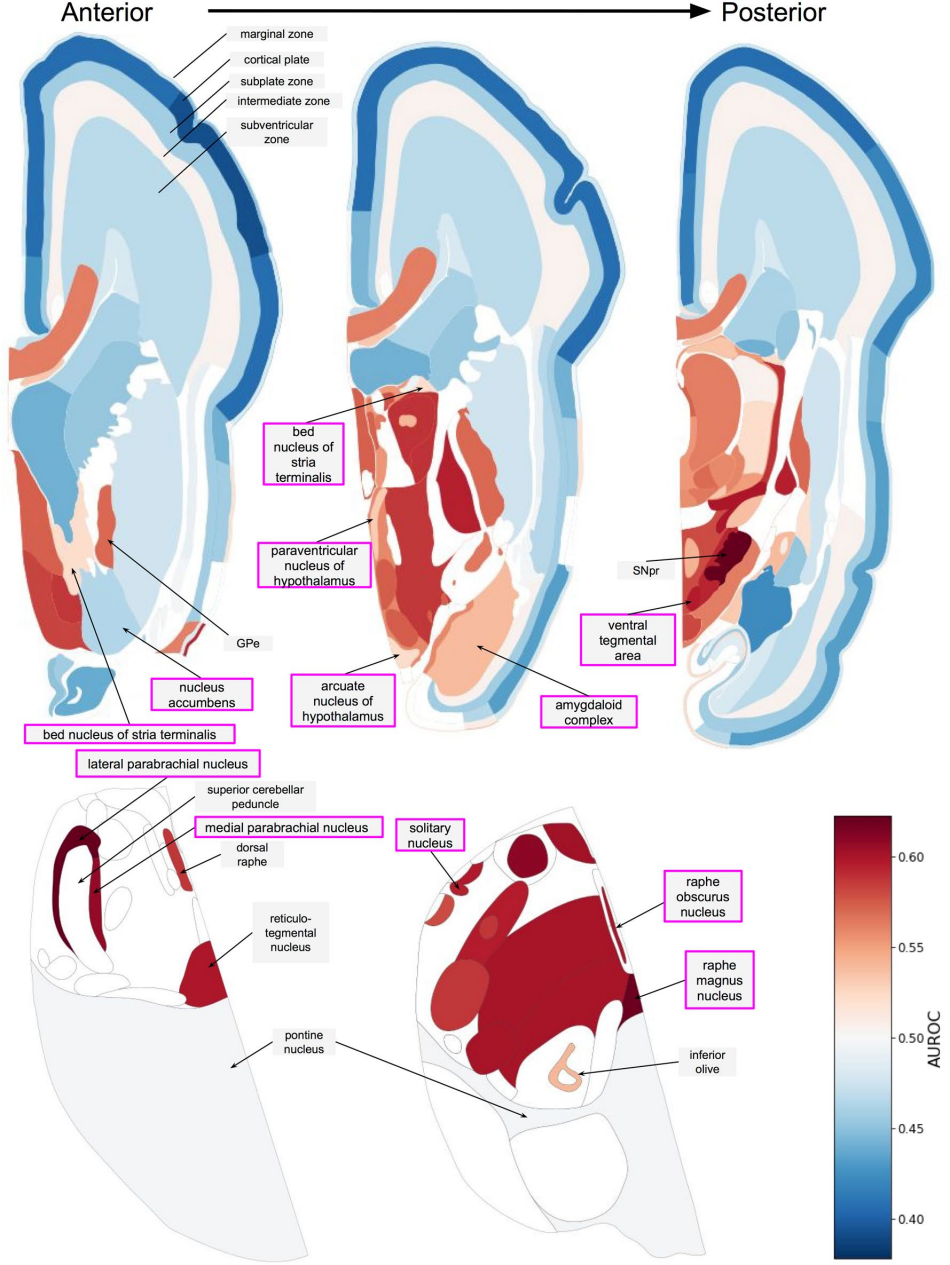
Anatomical maps showing aggregate gene expression patterns of the AN associated genes from Negraes et al. in the fetal brain. Regions of interest are highlighted with purple boxes. AUROC values range from depleted expression in dark blue to enriched in dark red with missing values in white. (GPe: globus pallidus external segment; SNpr: substantia nigra pars reticulata).

We first characterized the expression pattern of the largest set of genes, which were differentially expressed in iPSC-derived neurons from anorexia nervosa patients in comparison to controls^21^. We used the adult and prenatal human gene expression data from the Allen Brain atlases to evaluate brain-wide expression patterns. For the 289 genes that remained after integration, our analysis identified 40 of 232 brain regions in the adult brain and 128 of 516 fetal brain regions where expression of the Negraes genes was higher than expected (all AUROC > 0.538, p_FDR_ < 0.05, Supplement Tables 3 and 4). The top region in the adult analyses was the lateral parabrachial nucleus (AUROC = 0.579, p_FDR_ < 10^–4^), followed by the pontine raphe nucleus (AUROC = 0.574, p_FDR_ < 0.0005). While no regions of the cerebral cortex were enriched after brain-wide multiple test correction, the most enriched cortical area was the lateral bank of the parahippocampal gyrus (AUROC = 0.525, p_FDR_ > 0.3). In the prenatal brain, the lateral parabrachial nucleus and the raphe magnus nucleus rank second and third respectively within the 516 fetal regions (AUROC > 0.62, p_FDR_ < 10^–9^). Because these regions have been previously associated with feeding behaviour in mice and rats, we focused our analyses on several regions of interest. These regions were selected from circuits previously associated with feeding in rodents or have been targeted by deep brain stimulation for the treatment of anorexia nervosa (or proposed targets; see Methods). In the adult expression data, 5 of the 11 regions of interest preferentially expressed the Negraes genes after correction for the 232 tested regions (pontine raphe nucleus, raphe nuclei of medulla, ventral tegmental area, lateral and medial parabrachial nucleus; all p_FDR_ < 0.001; Figs. 2 and 3). In the fetal brain, these regions were also enriched for higher expression of the Negraes genes after brain-wide multiple test correction (all p_FDR_ < 10^–6^, Figs. 2 and 4). The solitary nucleus (AUROC = 0.595, p_FDR_ < 10^–6^), and the central amygdala (AUROC = 0.549, p_FDR_ < 0.05) are additionally enriched with the subgenual cingulate cortex having depleted expression (AUROC = 0.425, p_FDR_ < 0.0001). Overall, genes that are differentially expressed in neurons derived from anorexia nervosa patients are strongly expressed in feeding circuits and is consistent in both the adult and fetal brain.

### Watson GWAS gene lists: regional enrichment tests

The above genes were obtained from a small study of anorexia nervosa cases and obtained from induced neurons. As a result, the associated regional enrichment may be driven by experimental variables or may not generalize to a broader set of cases. To overcome these limitations, we examined genes from the largest GWAS of anorexia nervosa to date that associated 8 loci with anorexia nervosa^24^. This study of 16,992 cases, provides genetic associations that are not apriori linked to a tissue of interest.

We evaluated expression enrichment in the regions of interest for the 107 protein-coding genes annotated as proximal to the 8 genome-wide significant loci (105 genes with available data). In the adult human brain, we observe the most specific enrichment of the protein-coding genes in the arcuate nucleus of the hypothalamus, this subcortical structure is ranked 1st of all 232 brain regions and survives brain-wide correction (AUROC = 0.60, p_FDR_ < 0.015, Supplement Table 5). None of the other regions of interest were enriched for expression of these genes. When using only the protein-coding genes proximal to the first locus, the arcuate nucleus of the hypothalamus is again ranked first (AUROC = 0.60, p_FDR_ < 0.035). When using the MAGMA derived gene list, we observe similar but weaker enrichments. Across our regions of interest, the subcallosal cingulate gyrus most specifically expresses MAGMA genes (ranked 3/232 brain-wide, AUROC = 0.61, p_FDR_ = 0.0501). Using the fetal expression data, no regions of interest are enriched after correction, for either gene list.

### Duncan GWAS gene list: regional enrichment tests

**Figure 5:**
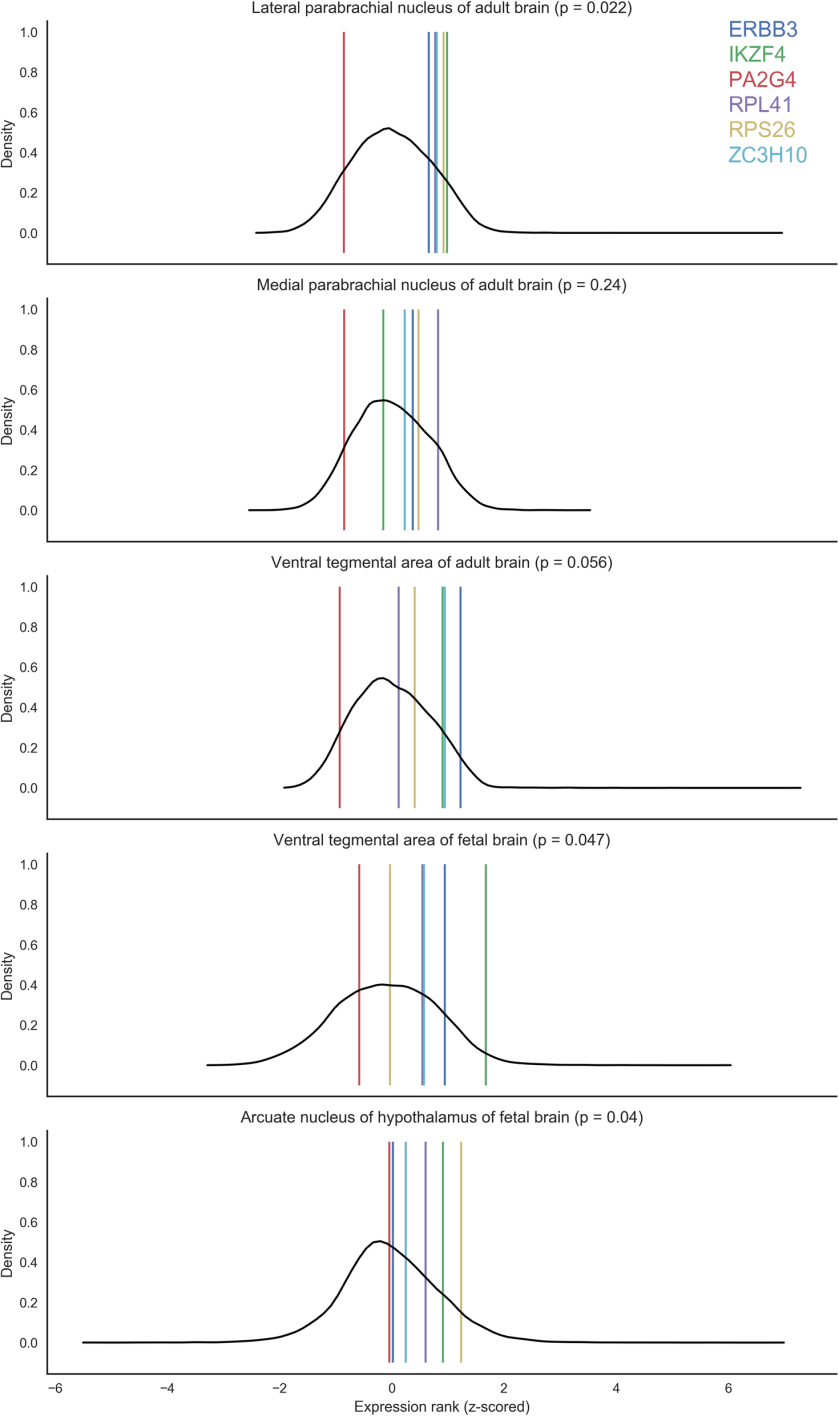
Density plots of z-scored genome-wide expression within a brain region for either the adult or fetal brain reference atlases (in black). Coloured lines mark expression of the 6 genes near rs4622308.

To provide another test of the regions identified from the Negraes list, we examined genes from the first GWAS of anorexia nervosa that identified loci with genome-wide significance (rs4622308). This locus overlaps with *IKZF4*, *RPS26*, *ERBB3*, *PA2G4*, *RPL41*, and *ZC3H10*^23^. For our AUROC analyses, the statistical power is driven by number of genes. For this smaller set of six genes, we only tested for expression enrichment of these genes in the regions of interest and report uncorrected p-values and brain-wide rankings. In the adult data, specific expression was observed in the lateral parabrachial nucleus (AUROC = 0.74, p_uncorrected_ = 0.022, brain-wide rank: 6 of 232) and a weaker signal in the ventral tegmental area (AUROC = 0.69, p_uncorrected_ = 0.056, brain-wide rank: 14). In the fetal data, specific expression was observed in the arcuate nucleus of the hypothalamus (AUROC = 0.71, p_uncorrected_ = 0.04, brain-wide rank: 37 of 516) and the ventral tegmental area (AUROC = 0.7, p_uncorrected_ = 0.047, brain-wide rank: 41). To visualize these enrichments, expression rankings of the six genes in these regions are marked in Fig. 5. Across the six genes within these regions, *PA2G4* shows consistent depleted expression. Broadly, this gene is expressed at lower levels in the subcortex with the highest expression in the posterior cingulate cortex. Of these three regions, all were brain-wide significant using the preceding Negraes or Watson lists, thus increasing the confidence of their involvement in anorexia nervosa.

### Lutter gene variant list: regional enrichment tests

In addition to the genes near common variants associated with anorexia nervosa, we assessed the patterns of genes that harbour novel and ultra-rare damaging variants in cases diagnosed with the restricting subtype ^22^. In support of previous findings, specific expression was observed in the lateral parabrachial nucleus in the adult brain data (AUROC = 0.58, p_uncorrected_ = 0.048, brain-wide rank: 9, Supplement Table 6). In the fetal brain, no regions reached significance, but all except the subgenual cingulate cortex had higher expression than average (AUROC > 0.5). We also note that the lateral parabrachial nucleus had the highest AUROC value of the fetal regions of interest tested (AUROC = 0.566, p_uncorrected_ = 0.10, brain-wide rank: 27 of 516). The second list of genes provided by Lutter et al., that harbour damaging variants in cases with binge-eating behaviour were not enriched in any of the brain regions of interest in the adult or fetal brains (all p_uncorrected_ > 0.05, obtained from patients with anorexia nervosa binge-eating/purging subtype, bulimia nervosa, and binge-eating disorder diagnoses). Overall, this further marks the lateral parabrachial and suggests subtype differences.

### Regional expression enrichment summary

To provide a high-level view of our anatomical results, we combined the findings from the four sources of anorexia nervosa associated genes and two anatomical expression atlases in Table 1. For specific lookups, regional expression profiles for each targeted gene is available in Supplemental Table 7. After filtering for brain-wide multiple test correction, the parabrachial, raphe, ventral tegmental, arcuate nucleus of the hypothalamus, solitary nucleus, and central amygdala regions are statistically significantly enriched for higher expression. Most of these are brain-wide significant in both adult and fetal datasets, except for solitary nucleus (not profiled in adult), arcuate nucleus of the hypothalamus, and central amygdala. When restricted to brain-wide significant regions with evidence at uncorrected thresholds, the lateral parabrachial nucleus (4 enrichment tests), ventral tegmental area (3 tests), and arcuate nucleus of the hypothalamus (2 tests) reoccurred. Overall, this summary view highlights agreements that cross fetal, adult, transcriptomic, and genetic perspectives.

**Table 1:** Summary of expression enrichment in the fetal and adult brain expression atlases (**denotes p_FDR_ < 0.05, + denotes p_uncorrected_ < 0.05, and - marks non-significant enrichment or depleted expression).

Brain region	Negraes		Watson protein-coding		Duncan		Lutter restricted eating		Lutter binge eating	
	Fetal	Adult	Fetal	Adult	Fetal	Adult	Fetal	Adult	Fetal	Adult
Lateral parabrachial nucleus	**	**	–	–	–	+	–	+	–	–
Medial parabrachial nucleus	**	**	–	–	–	–	–	–	–	–
Paraventricular nucleus of the hypothalamus	–	–	–	–	–	–	–	–	–	–
Arcuate nucleus of the hypothalamus	–	–	–	**	+	–	–	–	–	–
Pontine and midbrain raphe nuclei	**	**	–	–	–	–	–	–	–	–
Subcallosal or subgenual cingulate cortex	–	–	–	+	–	–	–	–	–	–
Nucleus accumbens	–	–	+	–	–	–	–	–	–	–
Ventral tegmental area	**	**	–	–	+	–	–	–	–	–
Central nucleus of the amygdala	**	–	–	–	–	–	–	–	–	–
Bed nucleus of stria terminalis	–	–	–	–	–	–	–	+	–	–
Solitary nucleus	**		–		–		–		–	

### Cell type marker enrichment

The preceding analyses were performed with transcriptomic profiles of bulk tissue that combine expression information across cell-types. To test for cell-type specific signatures, we next determined if the gene lists are enriched for cell-type markers obtained from mouse studies. Only the Negraes gene set had significant cell-type marker enrichment after correction for the number of cell-types tested. Specifically, between 8 and 9 genes overlapped with the microglia markers across the four regional lists (all, cortex, amygdala, and brainstem). Unlike the other NeuroExpresso cell-type markers, these regional microglia marker lists strongly overlap because they were derived from analyses of the same microglia expression profiles from whole-brain samples in the context of other region-specific cell-type profiles ^42^. Across the four microglia lists, marker genes from the brainstem had the lowest corrected p-value (9 of 137 genes overlap, hypergeometric test, p_FDR_ < 0.01). These genes are split between up- and down-regulated in the Negraes results, suggesting the signal is not due to different microglia proportions (5 down-regulated and 4 up-regulated, Supplement Table 8). While not significant, overlap with the separate lists of microglia activation and deactivation were 4 and 2 genes, respectively. In addition, the largest overlap for the Lutter restricted eating associated gene list was with the brainstem microglia activation markers (*IL17RA* and *SLA*, p = 0.03, p_FDR_ = 1). Two markers of microglia deactivation were in the Watson list (*CAMP*, *UBA7*, p = 0.11). Of the six genes in the Duncan set, only the *ERBB3* gene was a Neuroexpresso marker (for oligodendrocyte precursors in the cortex). The binge eating associated genes from the Lutter study did not show any clear enrichment (1–2 genes per cell type). Overall, we find more than the expected number of microglia marker genes in the Negraes gene list.

### Gene expression responses to food deprivation in mice

**Figure 6:**
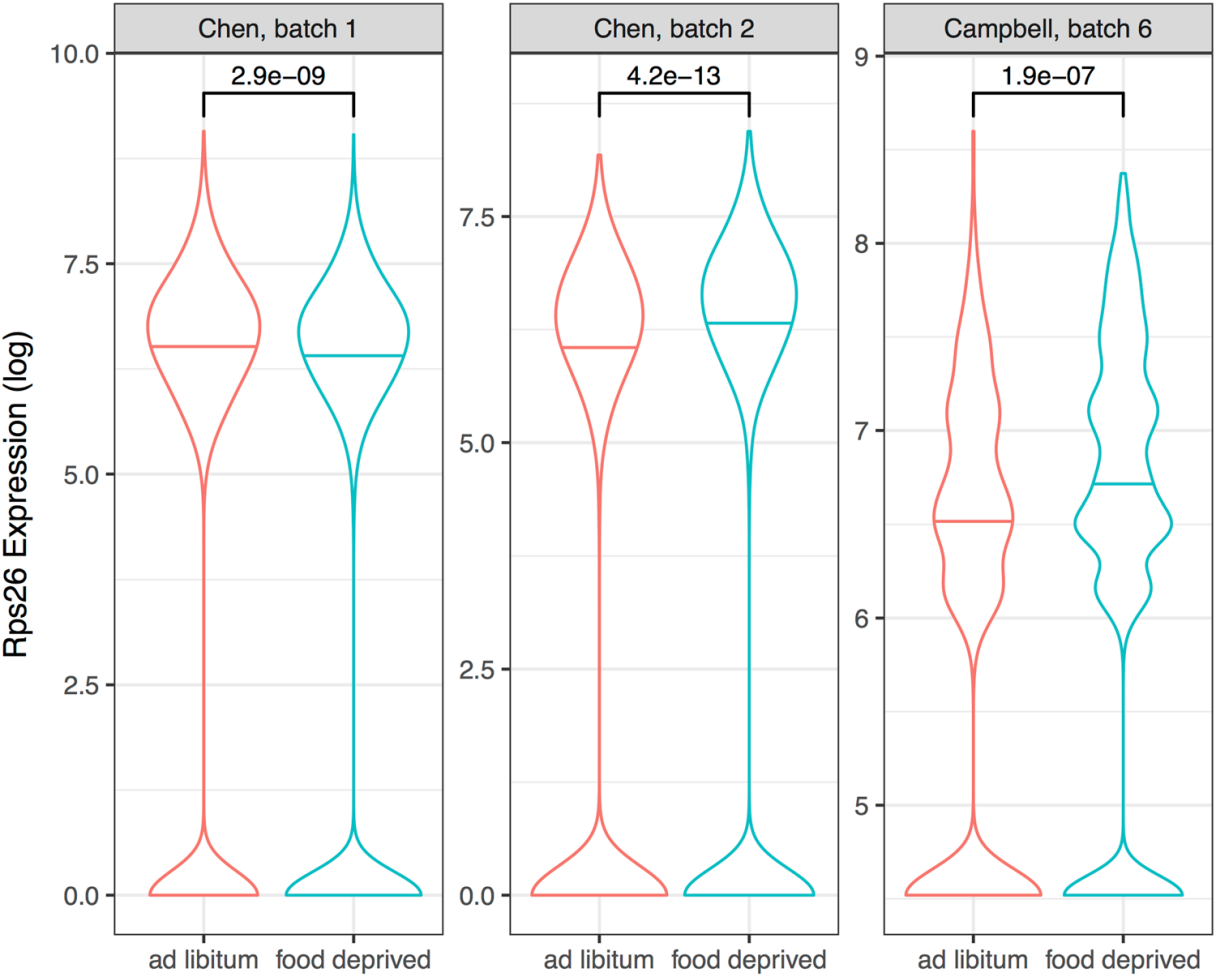
Violin plots of Rps26 single-cell expression in ad libitum (red) and food-deprived (blue) conditions. The first two plots are from the Chen dataset, where expression is measured by the log of transcripts per million. The last plot is from the Campbell dataset, which measured expression by the natural log of the counts per million plus one (unique molecular identifier method). Horizontal lines mark the median expression. Connecting brackets at the top provide p-values from Mann-Whitney U tests.

Motivated by enrichment of anorexia nervosa associated genes in subcortical appetitive circuits, we tested if expression of these genes is changed in fasted mice. We used two single-cell studies to determine if the genes of interest are differentially expressed as a result of 24 h of fasting in the female mouse hypothalamus (11,999 total cells) ^38,39^. Genome-wide, 2,160 of the 13,208 genes we examined were significantly differentially expressed (16.3%, p_FDR_ < 0.05). None of the gene lists were disproportionately enriched for these genes (Negraes: 17.6%, Watson: 14.1%, Duncan: 20%, Lutter restricted-eating: 20.6%, and Lutter binge-eating: 18.75%). In the Duncan set, *Rps26* was significantly up-regulated after fasting in both datasets (Fig. 6, meta-p_FDR_ < 10^–13^). However, this finding is not consistent with one batch of the Chen dataset showing down-regulation after fasting (meta-p_FDR_ < 10^–4^). In contrast, we did not detect any significant changes in expression for the other four genes with mouse homologs near rs4622308. While not significantly enriched, genes that are altered after fasting and associated with anorexia nervosa are of interest (full list provided in Supplement Table 9).

## Discussion

We first investigated the neuroanatomical expression patterns of genes associated with anorexia nervosa. We used a polygenic approach that does not require that all the associated genes are causal. Our results show that anorexia nervosa associated genes are highly expressed in regions linked to food intake and reward in rodent studies, suggesting direct relevance to human studies of anorexia nervosa. The most consistent region of enrichment is the parabrachial nucleus. In rodents, this region was once named the “pontine taste area”^43^ and plays a key role in appetite regulation. In mice, glutamatergic neurons in the dorsolateral parabrachial project to the substantia nigra, forming a key link in the gut-to-brain pathway^44^. Within the lateral parabrachial nucleus, calcitonin gene-related peptide (CGRP) neurons in the have been shown to inhibit feeding, suppress appetite^18^, be necessary and sufficient in the maintenance and expression of conditioned taste aversion^45,46^, control meal termination^19^, and prevent overeating^47^. More broadly, CGRP neurons in the parabrachial are thought to serve as a general-purpose alarm that encodes diverse danger signals^43,48^. The *CALCB* gene, which encodes the beta isoform of CGRP, is the 23rd most differentially expressed gene from the anorexia nervosa derived stem cell study^21^. Within the anorexia nervosa associated genes we studied, *CALCB* has the most specific expression in the medial parabrachial nucleus in both the adult and fetal human brain data and ranks 2nd and 4th in the fetal and adult lateral parabrachial nuclei respectively (Supplement Table 7). Mouse studies have also revealed that the lateral parabrachial contains thermosensory relay neurons and is involved in thermoregulation^49,50^, suggesting relevance of this region to the colder body surface temperatures observed in anorexia nervosa patients^51, 52^. Loss of inhibitory hypothalamic connections from the arcuate to the parabrachial region leads to the cessation of feeding and starvation^15–17^. The arcuate nucleus of the hypothalamus was also found to be significantly enriched using an independent set of anorexia nervosa associated genes. Rodent studies have shown that this region drives food intake when activated and is thought to drive food-seeking behaviour [reviewed in^53^]. In addition, we also found enrichment of the ventral tegmental area, which receives hypothalamic input from orexin neurons that have been linked to appetite and reward ^54^. Activation of orexin receptors in the ventral tegmental area promoted food intake in a hedonic feeding model^55^. While our results do not suggest disturbances in all regions in feeding associated regions, they do link anorexia nervosa to subcortical appetitive circuits.

While we note that two CGRP related genes are highly ranked, at a broader cell-type level, we found enrichment of microglia genes. This is found first in the Negraes stem cell-derived genes and, to a lesser degree, in the list of damaging variants that were associated with anorexia nervosa. In mice, stimulation of the innate immune system by activation of toll-like receptor 2 resulted in sickness behavior that included anorexia^56^ and aberrant agouti-related protein signalling in an anorexia mouse model was associated with microglial activation^57^. Linking the regional results, the lateral parabrachial and the ventral tegmental area are enriched for the Neuroexpresso microglia marker genes (adult expression data: AUROC > 0.62, p_FDR_ < 10^–5^). Relative to the other regions, the lateral parabrachial nor the ventral tegmental area rank in the top 20 regions that are most enriched for microglia markers in adult expression data. In contrast, these two regions rank in the top 5 when using the Negraes stem cell-derived genes, showing that the microglia enrichment cannot fully explain the regional results. Microglia are key contributors to sex differences in the brain from both structural and functional perspectives^58^. In addition, microglial sex differences have been linked to behavioural differences and pain hypersensitivity^59–61^. In contrast to our findings of microglia markers in the top genetic hits, analyses that use genome-wide genetic data highlight neuron-specific genes and not microglia markers^24,62^. This difference may be due to the use of genome-wide statistics in those recent studies. In summary, our results suggest microglia deserve more attention in anorexia nervosa research and that they may help explain the higher prevalence of anorexia nervosa in females.

Our use of transcriptomic and genetic resources that crosses species and development marked robust signals but limits finer interpretations. Developmentally, we observed regional enrichment in the prenatal and adult brain but lack expression data from the adolescent brain for our regions of interest. Three of our sources of anorexia nervosa associated genes were not specific to disorder subtypes, and there is no significant overlap between the sets. The limitations of the different sources may explain this. In the GWAS results, genes are associated through proximity to associated common variants which may not alter expression of the nearby genes. In addition, these common variants are of small effect. The Lutter et al. variants have stronger causal implications, but this study did not determine if the rare variants were de novo or acquired^22^. From the transcriptomic perspective, expression changes of genes identified in the induced neural stem model may be causes or consequences of the disorder. In contrast to the genetic studies, the stem cell models are limited in sample size, which reduces generalizability. Even across GWAS studies, heterogeneity is supported by the finding of cohort differences for the first identified genetic variant^23, 24^. Our use of several datasets, each with different limitations, helps provide converging insight into the molecular neuroanatomy of anorexia nervosa.

Our main gene list was from the Negraes et al. stem cell study. Their differentiation procedure generated primarily cortical neurons, with a low proportion of glial cells. Our findings of microglia and subcortical expression enrichment suggest follow-up study of the Negraes et al. differentially expressed genes should include microglia and subcortical regions. While providing a weaker signal, we validated enrichment of the microglia markers and the lateral parabrachial and ventral tegmental areas with genetically associated genes that are not inherently linked to a specific tissue or cell type.

Within the stem cell-derived gene list from Negraes et al., tachykinin receptor 1 (*TACR1*) was identified as a novel and potential contributor to anorexia nervosa pathophysiology^21^. In mice, noxious stimuli were found to activate tachykinin precursor 1 (*Tac1*) expressing neurons in the lateral parabrachial nucleus and trigger escape behaviour^63^. While lower in the fetal brain, in the adult data, *TACR1* has high expression in the lateral parabrachial nucleus (ranked 22^nd^ of 232 regions). In relation to the other anorexia nervosa associated genes, *TACR1* expression ranks 56th of 342 in the adult lateral parabrachial nucleus. When the three tachykinin related genes in the Negraes et al. genelist are combined (*TAC1*, *TACR1*, and *TACR2*), the lateral parabrachial nucleus is enriched in the adult data (ranked 7th of 232 regions, AUROC = 0.88, p_uncorrected_ = 0.02). This suggests that the proposed participation of the tachykinin system in anorexia nervosa involves the lateral parabrachial area.

A clear limitation of our study is the use of expression Atlases of the ‘normal’ human brain. Examination of postmortem brains of cases is possible, but it is difficult to determine which genes are perturbed by malnutrition or mark causal mechanisms. Due to these challenges, transcriptomic studies of anorexia nervosa that profile the brains of cases and controls are limited. A postmortem study by Jaffe et al. identified six differentially expressed genes in the prefrontal cortex of cases diagnosed with eating disorders (anorexia nervosa or bulimia nervosa) in comparison to controls^64^. While these six genes are not enriched in the lateral parabrachial or ventral tegmental areas, the most significant gene, *RFNG*, is strongly expressed in the adult lateral parabrachial nucleus (brain-wide: ranked 1st of 232 regions; genome-wide: 218 of 20,869 genes). The lack of more agreement with this study may be due to the mix of diagnoses, focus on the prefrontal cortex, or the effects of chronic illness in these postmortem samples. Predicting gene expression from genetic information avoids these pitfalls by using reference expression data from healthy subjects. This transcriptomic imputation approach was applied to loci identified in the Duncan et al. GWAS^65^. Within the rs4622308 locus, they identified 35 associations where differential expression was predicted for a specific gene and tissue. Across the six genes we used to represent rs4622308, they found significant associations for only *RPS26*. They also found that the predicted expression of *RPS26* was negatively correlated with BMI, weight, and waist circumference. In agreement, of these six genes, we also highlighted *Rps26* due to its differential expression in the hypothalamus of food-deprived mice. In the Watson et al. GWAS, genetic data was used to predict expression differences associated with the disorder. Among other genes, *DALRD3* expression was predicted to be lower in cases when using the tissue models for breast mammary tissue, esophagus mucosa, and skin not exposed suprapubic. We also highlight *DALRD3* because it’s mouse homolog is expressed at lower levels in the hypothalami of fasted mice (p_FDR_ < 0.0005). Here, we make use of single-cell data from mice to better understand the more rapid homeostatic changes in gene expression following fasting. It is important to note that such studies in mice of the effects of food restriction on gene expression in the hypothalamus only mimic some aspects of human anorexia nervosa. The use of mouse models of anorexia nervosa has shown some similarities to anorexia nervosa in humans with functional changes in appetitive circuits, feeding behaviour, energy expenditure, neuropeptide and hormonal alterations^66^. However, the lack of chronic models of food restriction limits the interpretations of human anorexia nervosa, which can endure for years and has a high relapse rate. Unlike our study, previous genomic studies did not highlight specific brain regions. This is probably due to the coarse resolution of the transcriptomic data that was limited to 13 large brain regions, which did not include the lateral parabrachial, arcuate nucleus of the hypothalamus, or ventral tegmental areas. While our results cannot be directly compared to past transcriptomic studies of cases and controls, we also mark *RPS26* and *DALRD3* as key genes in anorexia nervosa.

## Conclusion

In summary, we found that genes associated with anorexia nervosa are expressed at higher levels in the lateral parabrachial nucleus and the ventral tegmental area in comparison to the rest of the healthy adult and fetal brain. The adult expression enrichment of the lateral parabrachial is confirmed with genes from two independent genetic studies. In the fetal brain, enrichment of the ventral tegmental area is also observed for the six genes near the only common variant associated with the disorder (Table 1). We also observed signals in the adult and fetal pontine raphe, but they were not repeated when using the genes from the genetic studies. We also found more than the expected number of microglia marker genes in the anorexia nervosa associated genes. Finally, using mouse transcriptomic data, we noted several of the anorexia nervosa associated genes are differentially expressed during food deprivation. While these genes that respond to fasting are not enriched in the anorexia nervosa associated gene sets, we highlight *RPS26* and *DLALRD3*, which are proximal to common variants associated with anorexia nervosa. Our main finding of enriched expression in the lateral parabrachial nucleus suggests additional characterization of this region is warranted.

## Supplementary information

Supplementary Table 1

Supplementary Table 2

Supplementary Table 3

Supplementary Table 4

Supplementary Table 5

Supplementary Table 6

Supplementary Table 7

Supplementary Table 8

Supplementary Table 9
